# Hodgkin Lymphoma—The Effect of Chemotherapy on Gonadal Function and Fertility Is Strongly Related to the Treatment Regimen, Age, and Sex: A Systematic Review and Meta-Analysis

**DOI:** 10.3390/cancers18030425

**Published:** 2026-01-28

**Authors:** Mareike Roth-Hochreutener, Maria Karakitsiou, Angela Vidal, Susanna Weidlinger, Janna Pape, Tanya Karrer, Karolin Behringer, Michael von Wolff

**Affiliations:** 1 Division of Reproductive Endocrinology, University Hospital Zurich, 8091 Zurich, Switzerland; 2 Division of Gynecological Endocrinology and Reproductive Medicine, University Women’s Hospital, Inselspital Bern, University of Bern, 3010 Bern, Switzerland; maria.karakitsiou@insel.ch (M.K.); angela.vidal@insel.ch (A.V.); susanna.weidlinger@insel.ch (S.W.); janna.pape@insel.ch (J.P.); michael.vonwolff@insel.ch (M.v.W.); 3 Medical Library, University Library Bern, University of Bern, 3010 Bern, Switzerland; tanya.karrer@unibe.ch; 4 Hodgkin Study Group (GHSG), Department I of Internal Medicine, University Hospital Cologne, 50924 Cologne, Germany; karolin.behringer@uk-koeln.de

**Keywords:** Hodgkin disease, infertility, oncological treatment, fertility preservation, gonadal function, FertiTOX, FertiPROTEKT

## Abstract

**Background/Objectives**: Hodgkin lymphoma (HL) primarily affects individuals of reproductive age, making gonadal dysfunction after chemotherapy a critical survivorship concern. While fertility preservation options including gamete and gonadal tissue cryopreservation are available before treatment, evidence-based counseling requires regimen-specific risk estimates accounting for patient age and sex. Therefore, a meta-analysis was performed to assess presumed infertility in HL patients, stratified by chemotherapy regimen, age, and sex. **Methods**: This systematic review and meta-analysis, conducted within the FertiTOX project, included studies published between 2000 and February 2024. Eligible studies reported gonadal function outcomes ≥ 1 year after chemotherapy, excluding patients who received pelvic radiotherapy or stem cell transplantation, or had recurrent disease. Presumed infertility was defined by surrogate markers, including amenorrhea, premature ovarian failure, or abnormal hormonal levels in women, and azoospermia, oligozoospermia, or abnormal hormonal levels in men. **Results**: Of 2376 screened studies, 50 were included (meta-analysis: 43 studies; 5564 female and 1631 male patients). Overall presumed infertility prevalence was 21% in women (95% CI: 0.14–0.29) and 45% in men (95% CI: 0.29–0.62). The highest prevalence occurred after BEACOPP (women 38%; men 81%), while ABVD was associated with the lowest (6% each in women and men). Childhood/adolescent HL treatment resulted in lower prevalence in women (8%) but remained high in men (67%). **Conclusions**: Fertility risk depends on regimen, age, and sex, requiring tailored counseling. For female children/adolescents and all patients receiving ABVD, post-treatment fertility evaluation and treatment may suffice. However, pre-treatment fertility preservation is strongly recommended for male adolescents and patients receiving other regimens.

## Simple Summary

Hodgkin lymphoma (HL) mainly affects people during their reproductive years, making fertility an important part of survivorship. Although cure rates are high, chemotherapy can impair gonadal function, with the extent of damage depending on the specific regimens used, as well as on age and sex. To provide clearer, evidence-based counselling, we systematically reviewed studies published since 2000 and combined data from more than 7000 patients. Overall, the likelihood of presumed infertility after treatment was approximately 21% in adult women and 45% in adult men. Infertility risk varies widely by regimen: it is low after ABVD but substantially higher after regimens containing alkylating agents. Men are generally at greater risk than women, and boys treated during childhood/adolescence show particularly high vulnerability. These findings highlight the importance of personalized fertility counselling and help determine when fertility preservation before treatment is essential and careful post-treatment assessment may be sufficient.

## 1. Introduction

Hodgkin lymphoma (HL) represents a distinct malignancy of the lymphatic system, with peak incidence during the prime reproductive years (ages 15–35) [1]. This temporal overlap between disease onset and family planning poses critical challenges for the increasing number of survivors, particularly as incidence rates continue to rise in younger populations and among women [2]. Given current 5-year survival rates exceeding 95% in early-stage disease, fertility preservation has become a key quality-of-life consideration [3].

In early stages, patients between the ages of 18 and 60 years are commonly treated with two cycles of Adriamycin, Bleomycin, Vinblastine, and Dacarbazine (ABVD) followed by involved site radiotherapy (IS-RT), leading to 5-year survival rates of 97% [4,5]. In intermediate stages and among adults, combination chemotherapy, consisting of two cycles of Bleomycin, Etoposide, Adriamycin (doxorubicin), Cyclophosphamide, Oncovin (vincristine), Procarbazine, and Prednisone (BEACOPP), followed by two cycles of ABVD, is commonly administered. Treatment may be followed by involved site radiotherapy (IS-RT), with a reported 5-year survival rate of 99% [6]. In advanced stages, chemotherapy regimens such as BEACOPP and, more recently, Brentuximab vedotin, Etoposide, Cyclophosphamide, Doxorubicin, Dacarbazine, and Dexamethasone (BrECADD) are applied [7,8]. Treatment decisions are guided by interim staging using positron emission tomography/computed tomography (PET/CT), and both approaches are associated with high survival rates.

Chemo- and radiotherapy carry the risk of infertility. The effect of chemotherapy on gonadal function depends on the treatment regimen and the age of the patient and is different in females and males. In particular, the alkylating agents procarbazine and cyclophosphamide, are highly gonadotoxic [9,10,11].

For young HL patients, family planning is often not yet complete or has not yet been discussed at the time of initial diagnosis. Accordingly, around 20% of women counseled for fertility preservation measures are diagnosed with lymphoma, mostly HL [12].

To avoid under- or overtreatment in terms of fertility preservation measures, it is crucial to evaluate the potential risk of infertility prior to the initiation of gonadotoxic therapies. Many studies, including those conducted by the German Hodgkin Study Group (GHSG), have investigated the impact of different chemotherapies on gonadal function. Various parameters such as hormone levels, sperm analyses, and menstrual cycle history in women were used [7,13,14]. The knowledge provided by these studies needs to be consolidated by systematically and comprehensively analyzing and summarizing all studies published since 2000.

The objective of our systematic review and meta-analysis is to perform such a systematic and comprehensive summary and to provide a precise assessment of presumed infertility associated with different chemotherapies and the age of patients with HL. The study is part of the FertiTOX project (www.fertitox.com, accessed on 3 August 2025), which aims to close the data gap regarding the risk of infertility of anticancer therapies to provide more accurate advice about fertility preservation measures [15,16,17,18,19,20,21,22]. It serves as a comprehensive reference for oncologists and reproductive physicians to inform their patients regarding the advisability of fertility preservation prior to chemotherapy.

## 2. Materials and Methods

### 2.1. The Registration of Protocols

The protocol for this study was registered with the International Prospective Register of Systematic Reviews (PROSPERO; registry number: CRD42023384052). The study was conducted in accordance with the Preferred Reporting Items for Systematic Reviews and Meta-Analyses (PRISMA) guidelines [23].

### 2.2. Search Strategy

**Figure 1:**
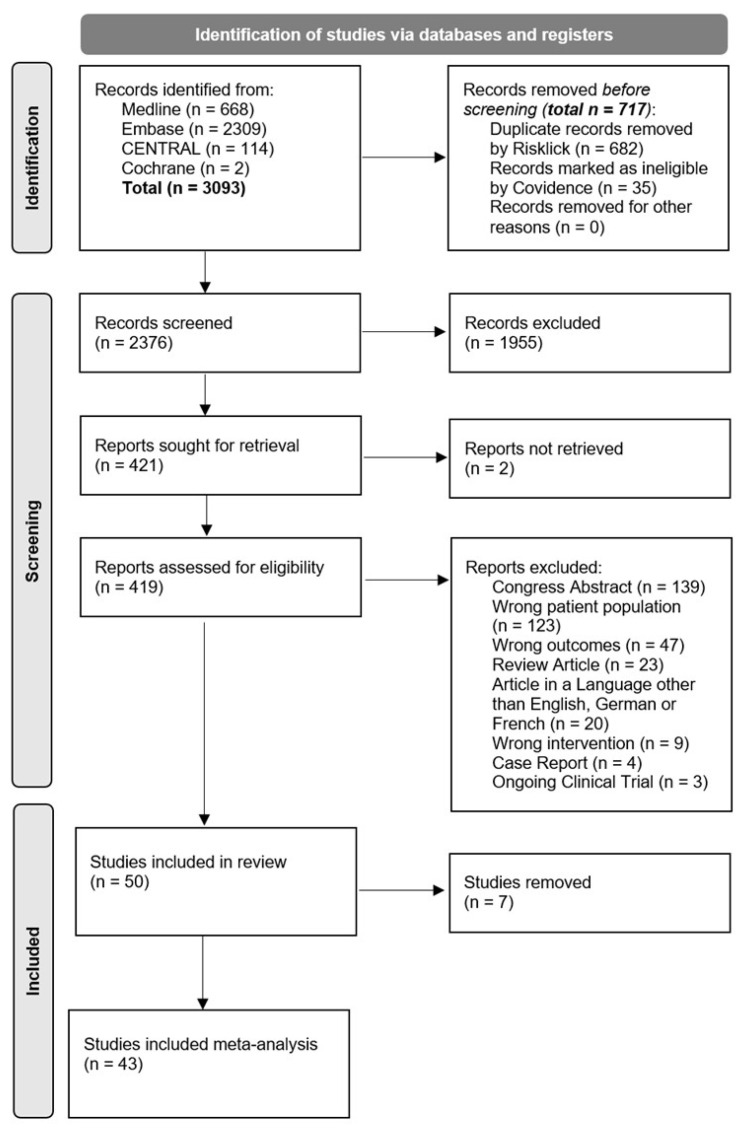
PRISMA flow diagram. A flowchart of the literature search and selection process.

A systematic literature search was conducted in February 2024 across MEDLINE, Embase, the Cochrane Database of Systematic Reviews, and CENTRAL (Figure 1). An initial search strategy for Embase was developed by an experienced medical librarian and validated against a test reference set. Following refinement and consultation, tailored search strategies were constructed for each database using a combination of controlled vocabulary (e.g., thesaurus terms and subject headings) and free-text terms, including synonyms, acronyms, and related expressions. The search was restricted to publications from 2000 to February 2024 and encompassed all subtypes of HL.

A double-negative strategy incorporating the Ovid “humans-only” filter was applied to exclude animal-only studies. Detailed search strategies for each database are available in Supplementary File S1. In addition to database searches, the reference lists of relevant articles and reviews were manually screened to identify further eligible studies. All retrieved citations were imported into Covidence and duplicate records were identified and removed [24].

### 2.3. Inclusion and Exclusion Criteria

Study selection was performed independently by three reviewers (MR, MK, AV) using Covidence (www.covidence.org, accessed on 3 August 2025) [25]. Original articles were included if they reported on HL, treatment modality (at least treatment with chemotherapy), and fertility outcomes measured at least one year after treatment cessation, with the provision that sufficient data was available to calculate prevalence estimates. Presumed infertility, the primary outcome of this study, was defined as reproductive impairment based on surrogate markers rather than confirmed live birth outcomes. Table 1 outlines the specific clinically relevant surrogate markers used to define presumed infertility in this meta-analysis. Studies in which presumed infertility could not be assessed according to these predefined criteria were excluded. For studies reporting the parameters listed in Table 1 in a non-extractable form or only as median/mean values, corresponding authors were contacted to obtain original data. Studies were excluded if the reported mean or median age of female participants at diagnosis or treatment was 30 years or older. Studies with mean or median ages below 30 years were included even if they contained some patients aged 30 years or older, as individual-level exclusion was not feasible in aggregated data. The age threshold of 30 years was chosen to minimize confounding by age-related decline in ovarian reserve, which becomes increasingly relevant from the thirties onward and may independently affect fertility outcomes. This approach aimed to better isolate chemotherapy-associated gonadotoxicity from physiological age-related fertility decline. Patients who had undergone pelvic radiation or stem cell transplantation, as well as those receiving recurrence therapy, were not considered if they represented more than 10% of the total cohort.

### 2.4. Data Extraction

Data were extracted and independently reviewed by two investigators (MR and MK). The primary variables of interest included study population characteristics such as age at diagnosis and at outcome assessment, duration of follow-up, type of oncological treatment (especially chemotherapy regimens), and parameters used to define presumed infertility (Tables 2–5). Any discrepancies were resolved through discussion and consensus. If multiple surrogate markers of presumed infertility (as listed in Table 1) were available, the most unfavorable outcome was used. Chemotherapy regimens were grouped as all regimens combined, ABVD only, BEACOPP (including all regimens containing BEACOPP irrespective of cycle number, dose, or whether baseline or escalated), and other regimens. For studies lacking specific treatment details, it was assumed that patients had received chemotherapy.

### 2.5. Quality Assessment

The methodological quality of each included study was assessed using the Joanna Briggs Institute Critical Appraisal Checklist for Cohort Studies [27]. Each item was rated as “yes” (1 point), “no” (0 points), “unclear” (0 points), “partly” (0 points), or “not applicable” (0 points). Based on the total score, studies were classified as high quality (9–11 points), moderate quality (6–8 points), or low quality (0–5 points). Two authors (MR and MK) independently evaluated the risk of bias for each study. Any discrepancies were resolved through discussion and consensus. The quality ratings for all included studies are presented in Table 6.

### 2.6. Data Synthesis

The primary outcome of this systematic review and meta-analysis was the prevalence of presumed infertility in male and female patients with HL after chemotherapy. Subgroup analyses were conducted according to sex, chemotherapy regimen, and age at the time of treatment. To estimate prevalence, the number of individuals meeting the criteria for presumed infertility was divided by the total number of patients at risk, as reported in the respective studies. For pooled prevalence estimates, statistical analyses were performed using the “metafor” package in R (version 4.2.3; R Core Team, Vienna, Austria, 2013). Heterogeneity across studies was assessed using Cochran’s Q test and the I^2^ statistic. In cases of substantial heterogeneity, random-effects models were applied.

## 3. Results

### 3.1. Results of the Systematic Review

In total, 419 studies were assessed following screening of titles, abstracts, and full texts. Of these, 368 were excluded as they did not meet the predefined inclusion criteria, resulting in 50 studies being included in the systematic review and 43 in the meta-analysis (Figure 1).

### 3.2. Study Characteristics

The characteristics of the 43 studies included in the meta-analysis are presented in Table 2, Table 3, Table 4 and Table 5, stratified by adult females, adult males, female children/adolescents, and male children/adolescents. The studies comprised retrospective (n = 21), prospective (n = 19), and cross-sectional (n = 4) designs. The reviewed studies reported relevant outcomes used to define presumed infertility, as summarized in Table 1. In females, premature ovarian insufficiency or menstrual cycle disorders were the most commonly assessed outcomes, followed by abnormal hormonal parameters (elevated follicle-stimulating hormone (FSH), undetectable anti-Müllerian hormone (AMH)). In males, presumed infertility was predominantly defined by azoospermia or oligozoospermia, with some studies also applying elevated FSH levels. Methodological quality assessment classified 22 studies as high, 18 as moderate, and 3 as poor (Table 6). The studies originated from Europe (n = 34), Asia (n = 3), the United States (n = 2), Canada (n = 3), and South America (n = 1).

Sample sizes ranged from 3 to 1647 patients (adult females: 19–1647; adult males: 3–708; female children/adolescents: 5–73; male children/adolescents: 5–45). At diagnosis or therapy, the pooled median age was 27 years in both adult females (range 9–49) and adult males (range 6–69). Among female children/adolescents, the pooled median age was 13 years (range 3.8–18.8), and among male children/adolescents 11 years (range 0.1–19). Pubertal status was inconsistently reported: in female children/adolescents, most were post-menarchal, whereas in male children/adolescents a substantial proportion were prepubertal (up to 63%). At study participation, median age was infrequently reported, with pooled medians of 32 years in adult females (range 25–49), 28 years in adult males (range 27–49), and 23.3 years in children/adolescents (range 17.8–29). The pooled median follow-up was 7.4 years (range 1.5–20). Most studies specified the applied chemotherapy regimens; in four studies chemotherapy was reported without regimen details, and in three studies no treatment information was provided, with chemotherapy being assumed.

**Table 2:** Characteristics of the included studies, females (adults).

First Author, Year of Publication	Country	Study Design	Number of Participants of Interest (Females, Adults)	Age of Participants of Interest at Diagnosis/Therapy (Years, Range)	Age (Years, Mean +/− SD) at Outcome/ Evaluation	Follow-Up After Treatment, Length in Years (Range)	Tumor Type	Chemotherapy, Details	Infertility as Assumed	Infertility Parameter	Comments
Behringer et al. 2005 [13]	Germany	Retrospective	219	16–40 (60.5% < 30 years)	Not specified	3.2 (0.58–6.3)	HL, HD7: early stages (14.3%), HD 8: inter-mediate stages (46.4%), HD9: advanced stages (39.3%)	HD7: Arm A: no chemotherapy (excluded), Arm B: ABVD; HD8: Arm A: COPP/ABVD, Arm B: COPP/ABVD; HD9: Arm A: COPP/ABVD, Arm B: standard BEACOPP, Arm C: increased-dose BEACOPP	76/219 (34.7%) *, 1/9 (11.1%) ▲, 25/116 (21.6%) ◦, 50/94 (53.2%) □	* Calculated in women with secondary amenorrhea, all chemotherapy regimens; ▲ ABVD; ◦ other chemotherapies (COPP/ABVD); □ BEACOPP	
Verschuuren et al. 2006 [28]	The Netherlands	Retrospective	48	27 (18–44)	Not specified	10 (1.1–15)	HL, stages I and II	ABVD, EBVP, MOPP/ABV, BEACOPP	8/48 (16.7%) *	* Calculated in women with POF (no spontaneous menstrual cycle within 5 years after antitumor treatment), all chemotherapy regimens	
Haukvik et al. 2006 [29]	Norway	Retrospective	67	Complete cohort (N = 99 patients; N = 67 with chemotherapy, N = 32 without chemotherapy): POF-group: 27 (17–39), Non-POF-group: 25 (9–39)	44	20 (10–25)	HL, Ann arbor stage I: 18.7%, stage II: 47.7%, stage III: 22.4%, stage IV: 11.2%	ABOD or EBVP (no alkylants): 19.4%, MVPP or ChlVPP (alkylants): 80.6%	33/67 (49.3%) *, 3/13 (23.1%) ▲, 30/54 (55.6%) ◦	* Calculated in women with POF (amenorrhea < 41 years, after other possible causes for amenorrhea have been excluded; no FSH values available), all chemotherapy regimens; ▲ ABVD; ◦ other regimens (MVPP, ChlVPP)	
Giuseppe et al. 2007 [30]	Italy	Prospective	29	24.3 (+/−SD 6.6)	28.5 (± SD 7.3); <30 years: 69%, 30–36 years: 17.2%, 37–40 years: 13.8%	4.2 (+/−SD 2.8)	HL, Ann arbor stage I: 6.9%, stage II: 75.9%, stage III: 17.2%	MOPP/ABVD (44.8%), ABVD (44.8%), MOPP/ABVD/ DHAP (10.3%)	8/29 (27.6%) *	* Calculated in women with amenorrhea, all chemotherapy regimens	Co-treatment with GnRH-a: 14/29 (48%): all with amenorrhea in non-GnRH-a group; no significant difference in hormonal values (AMH, FSH, LH, Inhibin B) between GnRH-a/no GnRH-group
Huser et al. 2008 [31]	Czech Republic	Prospective with historical controls	117	30.4 (18–35)	Not specified	0.5–1	Newly diagnosed HL	Group A: ABVD, Group B: ABVD+ BEACOPP (4 cycles), Group C: BEACOPP (8 cycles)	47/117 (40.2%) * 14/39 (35.9%) ▲ 33/78 (42.3%) □	* Calculated in women with POF (FSH > 15 IU/L and no regular bleeding, 1 year after end of treatment), all chemotherapy regimens, ▲ ABVD, □ BEACOPP	Case group (N = 72): Chemotherapy plus GnRH analogues; Control group (N = 45): same chemotherapy protocol but without GnRH analogues; POF rate in case group: 20.8%, POF rate in control group: 71.1%
Blumenfeld et al. 2008 [32]	Israel	Prospective	111 (GnRH-a/Chemotherapy group: N = 65; Chemotherapy (similar regimens) only group: N = 46)	GnRH-a/Chemotherapy group: 23.96 (SD +/− 5.47); Chemotherapy only group: 25.46 (SD +/− 6.41)	Not specified	8 (2–15)	Classical HL, mostly stages II–III	ABVD, BEACOPP, MOPP-ABV(D)	19/111 (17.1%) *, 1/35 (2.9%) ▲, 7/37 (18.9%) □, 11/39 (28.2%) ◦	* Calculated in women with POF (hypergonadotropic amenorrhea: FSH > 40 U/L on at least 2 occasions and low E2 levels), all chemotherapy regimens; ▲ ABVD, □ BEACOPP, ◦ other regimens (MOPP-ABV(D))	Pregnancies (spontaneous conception): GnRHa-/CT group: N = 26, CT only group: N = 20, all healthy neonates, no congenital anomalies; POF incidence was significantly lower in the GnRHa group than in the chemotherapy-only group
De Bruin et al. 2008 [33]	The Netherlands	Retrospective	276	Complete cohort (N = 518): 25 (14–39); Subdivision in age groups: 14–21: 33.2%; 22–28: 32.8%; 29–39: 34.0%; none of the women were treated before menarche	Not specified	9.4	HL, complete cohort (N = 518): Stage I: 17.8%, II: 52.7%; III: 12.4%; IV: 6.9%; unknown: 10.2%	ABVD, EBVP, others (MOPP, MOPP/ABV)	78/276 (28.3%) *, 6/50 (12%) ▲, 72/226 (31.9%) ◦	* Calculated in women with premature menopause (cessation of menses < age 40 years), all chemotherapy regimens; ▲ ABVD + EBVP; ◦ other therapies (MOPP, MOPP/ABV)	
Nitzschke et al. 2009 [34]	Switzerland	Cross-sectional	20	Not specified	25.1 (17–35)	Group A: 2.55 (1.55–3.75), Group B: 2.68 (0.5–4.83)	HL, Group A (N = 10): chemo-, radiotherapy and GnRHa; Group B (N = 10): similar chemotherapy but no GnRHa	ABVD, BEACOPP-14/s, OPPA + COPP/s	4/20 (20%) *, 0/12 (0%) ▲, 2/2 (100%) □, 2/6 (33.3%) ◦	* Calculated in women with amenorrhea, FSH > 10 U/L and/or AMH not detectable, all chemotherapy regimens, ▲ ABVD, □ BEACOPP, ◦ other regimens (OPPA/COPP/s)	No significant difference between Group A and B (GnRHa use or not) in terms of AMH, Inhibin B, FSH, and amenorrhea
Behringer et al. 2010 [35]	Germany	Prospective	19	25.6 (18–40)	Not specified	1.52 (1.04–2.78)	Advanced stage HL with risk factors	BEACOPPesc	18/19 (94.7%) *	* Calculated in women with AMH not detectable, FSH > 10 U/L or amenorrhea 12 months after the end of treatment	Random assignment to receive daily oral contraception (OC) or GnRH-a; Study closed prematurely due to no protection of the ovarian reserve with hormonal co-treatment (OC or GnRHa) during BEACOPPesc
Dann et al. 2011 [36]	Israel	Prospective	36	27 (18–37)	Not specified	7.42 (0.42–12)	Classical HL with adverse prognostic factors; Stages I/II: 35.8%; Stages III/IV: 64.2%	Escalated BEACOPP or standard BEACOPP	2/36 (5.6%) *	* Calculated in women with POF	Two patients with POF treated with GnRH-analogues (triptorelin); patient 1: stBEACOPP × 6; patient 2: stBEACOPP × 2 + eBEACOPP × 4
Behringer et al. 2012 [37]	Germany	Prospective	40	18–29	Not specified	HD-14 Arm A: 3.5 +/− SD 1.67 (1–6.92); HD-14 Arm B: 3.58 +/− SD 1.58 (1–6.42)	HL, stages IA, IB, IIA or IIB with ≥1 risk factor	HD-14 Arm A: ABVD; HD-14 Arm B: BEACOPPesc + ABVD (2 + 2)	3/40 (7.5%) *, 1/18 (5.6%) ▲, 2/22 (9.1%) □	* Calculated in women with FSH > 10 U/L, all chemotherapy regimens; ▲ HD-14 Arm A: ABVD, □ HD-14 Arm B: BEACOPPesc + ABVD	Pregnancies: HD-14 Arm A: 13%, HD-14 Arm B: 22%; Births: HD-14 Arm A: 12%, HD-14 Arm B: 13%
Van der Kaaij et al. 2012 [38]	The Netherlands	Retrospective	353	15–39 (15–24: 42%; 25–34: 42%, 35–39: 73%)	Not specified for POI evaluation group; 49 (25–76) for complete cohort	15 (5–45)	HL, Ann-Arbor stages I/II: 89%; III/IV: 11%	Nonalkylating agents: ABVD, EBVP; Alkylating agents: MOPP (6%), MOPP/ABV (33–36%), stBEACOPP (4%)	73/353 (20.7%) *, 4/151 (2.6%) ▲, 69/202 (34.2%) ◦	* Calculated in women with POF (defined as menopause before age of 40 years), all chemotherapy regimens; ▲ ABVD + EBVP; ◦ MOPP, MOPP/ABV, stBEACOPP	Live births: Women with POF: 22% with ≥1 children after treatment (age at treatment: n = 9: <25 years; N = 8 25–32 years; Women without POF: 41% with ≥1 children
Behringer et al. 2013 [14]	Germany	Prospective	90	Complete cohort: 28 +/− SD 7 (18–39), Suspected infertility subanalysis: 18–29	Complete cohort: 32 +/− SD 7 (20–45)	3.8 (1–8)	HL, HD 13: early favorable stage, HD14: early unfavorable/intermediate stage, HD15: advanced-stage	HD 13: ABVD or AVD; HD14: Arm A: ABVD; Arm B: BEACOPPesc + ABVD (2 + 2); HD15: escalated BEACOPP or BEACOPP-14	23/90 (25.6%) *, 1/25 (4%) ▲, 22/65 (33.8%) □	* Calculated in women with FSH > 10 U/L and without hormonal contraception at the time of the study, all chemotherapy regimens; ▲ ABVD or AVD, □ BEACOPP	Birth rates: HD13: 7% (1/15), HD14 Arm A: 14% (6/43), HD14 Arm B: 13% (6/48), HD15 Arm A: 5% (2/38), Arm B: 12% (3/26), Arm C: 4% (1/27)
Swerdlow et al. 2014 [39]	United Kingdom	Retrospective	906	Not specified, Study inclusion criteria: Age < 36 years, Age at first treatment: 0–14: 5.5%; 15–19: 20.7%, 20–24: 29.2%, 25–29: 24.3%, 30–35: 20.2%	Not specified	17.8 (0.3–48.4)	HL	ABVD, ChlVPP, LOPP, MVPP, MOPP	351/906 (38.7%) *, 2/144 (1.4%) ▲, 349/862 (49.5%) ◦	* Calculated in women with menopause < 40 years, all chemotherapy regimens, no or <10% with pelvic radiotherapy (RT); ▲ ABVD, no pelvic RT; ◦ other regimens (alkylants, ChlVPP, LOPP, MVPP, MOPP) with no or <10% pelvic RT	
Huser et al. 2015 [40]	Czech Republic	Prospective	108	27 (18–40)	Not specified	1–2	HL, Group A: Ann-Arbor Stage IA, IB, IIA or IIB without GHSG risk factors; Group B: Stages IA, IB, IIA or IIB with ≥1 GHSG risk factors; Group C: advanced HL (Stages III and IV or stage IIB with extranodal disease or large mediastinal mass)	Group A: ABVD; Group B: ABVD + BEACOPP (2 + 2); Group C: escBEACOPP	19/108 (17.6%) *, 2/44 (4.5%) ▲, 17/64 (26.6%) □	* Calculated in women with FSH ≥ 15 IU/L (defined as chDOR (chemotherapy induced diminished ovarian reserve) according to the study, one year after the end of chemotherapy, all chemotherapy regimens; ▲ ABVD; □ BEACOPP	Monthly triptorelin i.m. for all patients; Pregnancy achievement: within 2 years follow-up N = 23 (21.3%); no protection of the ovarian follicle pool with GnRH-a treated with regimen C (escBEACOPP) in this study
Boltezar et al. 2016 [41]	Slovenia	Retrospective	76	ABVD group: 26 (18–39), BEACOPP group: 26.5 (20–36)	Not specified	9 (2–16)	HL, ABVD group: Stages I: 4.3%, II: 84.8%, III: 2.2%, IV: 8.7%; BEACOPP group: I: 0%, II: 33.3%, III: 16.7%, IV: 50%	ABVD, BEACOPP	16/76 (21.1%) *, 3/46 (6.5%) ▲, 13/30 (43.3%) □	* Calculated in women with secondary amenorrhea, all chemotherapy regimens; ▲ ABVD; □ BEACOPP	Number of patients attempting conception post-treatment: ABVD group: 91.3%, BEACOPP group: 86.7%; Having children after treatment: ABVD: 45.2%, BEACOPP: 34.6%, *p* = 0.573
Anderson et al. 2018 [42]	United Kingdom	Prospective	364	ABVD-group: 26 (19–44), BEACOPP group: 31 (19–43)	Not specified	AMH measurements: 1–3; FSH measurements: 4.92 (3.49–5.08)	Classical HL, stages IIB-IV or IIA with adverse features	ABVD-AVD, BEACOPP	31/364 (8.5%) *, 13/339 (3.8%) ▲, 18/49 (36.7%) □	* Calculated in women with AMH not detectable and/or FSH > 25 U/L, all chemotherapy regimens; ▲ ABVD-AVD, □ BEACOPP	
Demeestere et al. 2021 [43]	Belgium, France	Prospective	66	Standard group: 25 (P25–P75: 22–31), Study group: 27 (P25–P75: 22–31)	Not specified	Checkup (hormones, sperm analyses) at baseline, end of treatment, and every year during 5 years of follow-up	HL, advanced stages	All patients: BEACOPPesc (2 cycles), Standard arm: additional BEACOPPesc (2 cycles), Study arm: if PET was negative: additional ABVD, if PET was positive: additional BEACOPPesc (2 cycles)	37/66 (56.1%) *	* Calculated in women with AMH not detectable, 10–14 months after end of treatment, all chemotherapy regimens	Pregnancy rates: no significant differences between study and standard group
Decanter et al. 2021 [44]	France	Prospective	87	Complete cohort (N = 122) with HL and non-HL, subdivision in AYA (adolescent and young adult) and Non-AYA; AYA group (15–24 years, 83% with HL): 20.7 ± 2.0; Non-AYA group (25–35 years, 68% with HL): 29.1 ± 3.2	Not specified	Serial AMH measurements from baseline up to 24 months after end of treatment	HL	ABVD, others (r-ACVBP, r-CHOP), BEACOPP	10/87 (11.5%) *, 1/65 (1.5%) ▲, 1/2 (50%) ◦, 8/20 □	* Calculated in women with AMH not detectable, 18 months after end of chemotherapy, all chemotherapy regimens; ▲ ABVD; ◦ others; □ BEACOPP	All patients received monthly GnRH agonist co-treatment during chemotherapy
Amzai et al. 2022 [45]	North Macedonia	Retrospective	81	27.3 (14–49)	Not specified	10	HL, complete cohort (N = 287, males and females): Stage I: 17%, II: 33.1%; III: 22.3%; IV: 25.8%; undefined: 1.8%	ABVD, BEACOPP	6/81 (7.4%) *, 4/74 (5.4%) ▲, 2/7 (28.6%) □	* Calculated in women with amenorrhea, all chemotherapy regimens; ▲ ABVD; □ BEACOPP	
Ciccarone et al. 2023 [46]	Italy	Prospective	68	28.0 (18–40)	Not specified	0.5–1	Classical HL, Stage I: 6.3%, Stage II: 56.9%, Stage III: 16.3%, Stage IV: 20.6%	ABVD, BEACOPP	11/68 (16.2%) * 11/51 (21.6%) ▲	* Calculated in women with AMH not detectable 12 months after end of treatment, all chemotherapy regimens; ▲ ABVD	51.1% of all patients undergoing chemotherapy received GnRHa during treatment
Flatt et al. 2023 [47]	Canada	Retrospective	1647	26 (+/−SD 6.7)	Not specified	Not specified (follow up until 40th birthday, end of the study (31 December 2018), bilateral oophorectomy, subsequent cancer diagnosis or death)	HL	Not specified	124/1647 (7.5%) *	* Calculated in women with POI (amenorrhea < 40 years, FSH > 25 IU/L)	
Luong et al. 2023 [48]	Canada	Retrospective	644	25.5 +/− SD 6.6 (15–39)	Not specified	Not specified (maximum follow-up 31/12/2019)	HL	Chemotherapy was given, but no information on regimens	33/644 (5.1%) *	* Calculated in women with POI	49% of patients had radiation therapy, but no details on radiotherapy localization available; POI risk in chemotherapy only group: 15/328 (4.6%), POI risk in chemotherapy + radiation group: 18/316 (5.7%)

Summary of cohort studies assessing the prevalence of infertility as assumed in adult females. The studies are sorted by year of publication. Age and duration of follow-up are given as years with mean (SD), or range where such data are available. Abbreviation: Diagnosis: HL Hodgkin lymphoma, HD Hodgkin disease, POF Premature Ovarian Failure, POI Primary Ovarian Insufficiency. Therapy: PET Positron Emission Tomography, RT Radiotherapy, GnRH-a Gonadotropin-Releasing Hormone agonist. Chemotherapy: ABOD Adriamycin (doxorubicin), Bleomycin, Oncovin (vincristine), Dacarbazine, ABV Adriamycin (doxorubicin), Bleomycin, Vinblastine, ABVD Adriamycin (doxorubicin), Bleomycin, Vinblastine, Dacarbazine, BEACOPP Bleomycin, Etoposide, Adriamycin (doxorubicin), Cyclophosphamide, Oncovin (vincristine), Procarbazine, Prednisone, stBEACOPP standard BEACOPP, BEACOPPesc. escalated BEACOPP, ChlVPP Chlorambucil, Vinblastine, Procarbazine, Prednisone, COPP Cyclophosphamide, Oncovin (vincristine), Procarbazine, Prednisone, DHAP Dexamethasone, High-dose Ara-C (cytarabine), Cisplatin, EBVP Etoposide, Bleomycin, Vinblastine, Prednisone, MOPP Mustine (mechlorethamine), Oncovin (vincristine), Procarbazine, Prednisone, MVPP Mustine (mechlorethamine), Vinblastine, Procarbazine, Prednisone, rACVBP reinforced Adriamycin (doxorubicin), Cyclophosphamide, Vindesine, Bleomycin, Prednisone, rCHOP—rituximab, Cyclophosphamide, Hydroxydaunorubicin (doxorubicin), Oncovin (vincristine), Prednisone. Parameters: AMH anti-Müllerian hormone, FSH follicle-stimulating hormone, LH Luteinizing Hormone. Others: GHSG German Hodgkin Study Group. * Presumed infertility parameter calculated after treatment with all chemotherapy regimens. ▲ Presumed infertility parameter calculated after treatment with ABVD. □ Presumed infertility parameter calculated after treatment with BEACOPP. ◦ Presumed infertility parameter calculated after treatment with other regimens.

**Table 3:** Characteristics of the included studies, males (adults).

First Author, Year of Publication	Country	Study Design	Number of Participants of Interest (Males, Adults)	Age of Participants of Interest at Diagnosis/ Therapy (Years, Range)	Age (Years, Mean +/− SD) at Outcome/ Evaluation	Follow-Up After Treatment, Length in Years (Range)	Tumor Type	Chemotherapy, Details	Infertility as Assumed	Infertility Parameter	Comments
Frias et al. 2003 [49]	USA	Prospective	3	27–40	28–42	1–2	HL	NOVP	1/3 (33.3%) *	* Calculated in men with azoospermia or oligozoospermia, treatment with NOVP	
Bizet et al. 2012 [50]	France	Retrospective	24	25 (+/−SD 7.6)	Not specified	4.1 (+/−SD 3)	HL	Not specified, treatment with chemotherapy assumed	12/24 (50%) *	* Calculated in men with azoospermia or oligozoospermia, treatment with chemotherapy assumed	
Van der Kaaij et al. 2007 [51]	The Netherlands	Prospective	293	30 (15–69)	Not specified	2.7 (1–11.3)	HL, Stage I: 32%, Stage II: 68%	ABVD, EBVP, MOPP, MOPP/ABV, BEACOPP	124/293 (42.3%) *, 8/101 (7.9%) ▲, 116/192 (60.4%) □	* Calculated in men with FSH > 10 U/L, all chemotherapy regimens; ▲ ABVD and EBVP; □ MOPP, MOPP/ABV, BEACOPP	
Sieniawski et al. 2008 [52]	Germany	Prospective	38	26 (16–58)	27 (16–52)	1.45 (0.08–7.8)	HL, Early stage: 11%, intermediate stage: 45%, advanced stage: 44%	ABVD, COPP/ABVD, BEACOPP baseline or BEACOPP escalated	71/103 (68.9%) *, 0/4 (0%) ▲, 37/59 (62.7%) ◦, 34/40 (85%) □	* Calculated in men with azoospermia, all chemotherapy regimens; ▲ ABVD; ◦other regimens (COPP + ABVD), □ BEACOPP	Median time to spermatogenesis recovery: 27 months
Kiserud et al. 2009 [9]	Norway	Cross-sectional	165	Complete cohort of HL and NHL patients: 33 (6–49)	Complete cohort of HL and NHL patients: 49 (21–73)	Complete cohort of HL and NHL patients: 15 (4–28)	HL, Stages I/II: 63.6%, Stages III/IV: 36.4%	ABVD/EBVP, OEPA, LVPP, BEACOPP, COPP, CHOP/COP	86/165 (52.1%) *	* Calculated in men with FSH > 12 U/L and/or low testosterone, all chemotherapy regimens	
Menon et al. 2009 [53]	France	Retrospective	6	17.81 +/− 0.14 (13–20)	27.36 +/− 1.23	4.5 +/− 0.68	HL	Treatment with polychemo-therapy combined with radiotherapy in 79% of cases	2/6 (33.3%) *	* Calculated in men with azoospermia, all therapies	
Behringer et al. 2013 [14]	Germany	Prospective	708	34 (18–49)	38 (19–57)	4 (1.5–4.1)	HL, HD 13: early favorable stage, HD14: early unfavorable/intermediate, HD15: advanced-stage	HD 13: ABVD or AVD; HD14: Arm A: ABVD; Arm B: BEACOPPesc + ABVD (2 + 2); HD15: escalated BEACOPP or BEACOPP-14	411/708 (58.1%) *, 34/200 (17%) ▲, 385/508 (75.8%) □	* Calculated in men with FSH > 10 U/L, all chemotherapy regimens; ▲ ABVD or AVD, □ BEACOPP	Birth after natural fertilization: After ABVD: 18/180 (10%), after BEACOPP: 6/472 (1.3%)
Tomlinson et al. 2015 [54]	United Kingdom	Retrospective and prospective; prospective arm concentrated on encouraging patients whose samples had been stored for a minimum of 18 months to attend for follow-up semen analysis	80	26 (13–49)	Not specified	3.33 (0.58–18.2)	HL	ABVD, other regimens (mainly MOPP, ChlVPP)	43/80 (53.8%) *, 3/28 (10.7%) ▲, 40/52 (76.9%) ◦	* Calculated in men with azoospermia, all chemotherapy regimens; ▲ ABVD; ◦ other regimens (MOPP, ChlVPP)	
Paoli et al. 2016 [55]	Italy	Retrospective	144	26 (13–51)	Not specified	Spermiogram at baseline (T0), 6 (T6), 12 (T12) and 24 (T24) months after end of treatment	HL	Group A: ABVD, Group B: escalated BEACOPP, COPP/ABVD, OPP/ABVD or MOPP	22/144 (15.3%) *, 0/115 (0%) ▲, 9/13 (69.2%) ◦, 13/16 (81.3%) □	* Calculated in men with azoospermia, all chemotherapy regimens; ▲ ABVD, 24 months after end of treatment, ◦ other regimens (ABVD/COPP, OPP, MOPP), 5–16 years after end of treatment, □ BEACOPP, 3–10 years after end of treatment	
Demeestere et al. 2021 [43]	Belgium, France	Prospective	43	Standard group: 29 (P25–P75: 23–36), Study group: 27 (P25–P75: 24–35)	Not specified	2.42 (2–2.92)	HL, advanced stages	All patients: BEACOPPesc (2 cycles), Standard arm: additional BEACOPPesc (2 cycles), Study arm: if PET was negative: additional ABVD, if PET was positive: additional BEACOPPesc (2 cycles)	40/43 (93%) *	* Calculated in men with azoospermia or oligozoospermia in standard and study group, 20–30 months after end of chemotherapy, all treated with BEACOPP	Pregnancy rates: higher likelihood of achieving pregnancy in PET-driven group (study group) (OR, 3.7; 95% CI, 1.4 to 9.3; *p* = 0.004)
Laddaga et al. 2022 [56]	Italy	Prospective	19	26 (15–37)	Not specified	8.7 (4.42–14.3)	HL, Stages I–II: 68%, Stages III–IV: 32%	ABVD	5/19 (26.3%) *	* Calculated in men with azoospermia or oligozoospermia, all treated with ABVD	Birth of child: N = 1/19 (5.3%), all treated with ABVD; no usage of cryopreserved semen
Amzai et al. 2022 [45]	North Macedonia	Retrospective	59	ABVD group: 24.8 (15–43); BEACOPP group: 29.2 (19–38)	Not specified	10	HL, complete cohort (N = 287, males and females): Stage I: 17%, II: 33.1%; III: 22.3%; IV: 25.8%; undefined: 1.8%	ABVD, BEACOPP	6/59 (10.2%) *, 1/49 (2%) ▲, 5/10 (50%) □	* Calculated in men with azoospermia or oligozoospermia, all chemotherapy regimens; ▲ ABVD; □ BEACOPP	

Summary of cohort studies assessing the prevalence of infertility as assumed in adult males. The studies are sorted by year of publication. Age and duration of follow-up are given as years with mean (SD), or range where such data are available. Abbreviation: Diagnosis: HL Hodgkin lymphoma, HD Hodgkin disease, NHL non-Hodgkin lymphoma Therapy: PET Positron Emission Tomography. Chemotherapy: ABVD Adriamycin (doxorubicin), Bleomycin, Vinblastine, Dacarbazine, AVD Adriamycin (doxorubicin), Vinblastine, Dacarbazine, BEACOPP Bleomycin, Etoposide, Adriamycin (doxorubicin), Cyclophosphamide, Oncovin (vincristine), Procarbazine, Prednisone, ChlVPP Chlorambucil, Vinblastine, Procarbazine, Prednisone, CHOP/OP Cyclophosphamide, Hydroxydaunorubicin (doxorubicin), Oncovin (vincristine), Prednisone/with or without Procarbazine, COPP Cyclophosphamide, Oncovin (vincristine), Procarbazine, Prednisone, EBVP Etoposide, Bleomycin, Vinblastine, Prednisone, LVPP Leukeran (chlorambucil), Vinblastine, Procarbazine, Prednisone, MOPP Mustine (mechlorethamine), Oncovin (vincristine), Procarbazine, Prednisone, NOVP Novantrone (mitoxantrone), Oncovin (vincristine), Vinblastine, Prednisone, OEPA Oncovin (vincristine), Etoposide, Prednisone, Adriamycin (doxorubicin). Parameters: FSH follicle-stimulating hormone. * Presumed infertility parameter calculated after treatment with all chemotherapy regimens. ▲ Presumed infertility parameter calculated after treatment with ABVD. □ Presumed infertility parameter calculated after treatment with BEACOPP. ◦ Presumed infertility parameter calculated after treatment with other regimens.

**Table 4:** Characteristics of the included studies, female children/adolescents.

First Author, Year of Publication	Country	Study Design	Number of Participants of Interest (Children, Females)	Age of Participants of Interest at Diagnosis/ Therapy (Years, Range)	Age (years, Mean +/− SD) at Outcome/ Evaluation	Follow-Up After Treatment, Length in Years (Range)	Tumor Type	Chemotherapy, Details	Infertility as Assumed	Infertility Parameter	Comments
Van den Berg et al. 2004 [57]	The Netherlands	Retrospective	14	Complete cohort of males and females (N = 76): MOPP group: 10.8 (5–14.3); ABVD group: 11.7 (3.8–15.2); ABVD/MOPP group: 13 (5–17.2)	Not specified	Complete cohort of males and females (N = 76): MOPP group: 16.3 (2–24.2); ABVD group: 12.3 (4.9–15.6); ABVD/MOPP group: 5.8 (0.6–11.3)	HL, Complete cohort of males and females: Stage I: 39.5%, Stage II: 26.3%, Stage III: 27.6%, Stage IV: 6.6%	MOPP, ABVD, MOPP/ABVD	2/14 (14.3%) *	* Calculated in women with irregular periods, all chemotherapy regimens	Pubertal status not mentioned
Gupta et al. 2016 [58]	Canada	Prospective	5	13.6 (12–14.3)	Not specified	1.67 (1.58–2.17)	HL	Cyclo-phosphamide, doxorubicin, cisplatin	0/5 (0%) *	* Calculated in women without menstruation resumption and/or AMH not detectable, all chemotherapy regimens	All patients were post-menarchal at the time of diagnosis/therapy
Drechsel et al. 2024 [59]	The Netherlands	Prospective	73	15.6 (7.3–18.8)	17.8 (IQR 15.8–19.1) at T4 (2 years post-diagnosis)	2 (IQR: 1.92–2.33)	Classical HL; early stage: 17.3%, intermediate stage: 48.1%, advanced stage: 34.6%	All treatment arms initially 2 cycles of OEPA induction treatment; Early stages: +1 cycle of COPDAC-28; intermediate stages: +2 cycles COPDAC-28 OR DECOPDAC-21; advanced stages: +4 COPDAC-28 OR DECOPDAC-21; 66% of the girls received COPDAC-28- and 34% received DECOPDAG-21	5/73 (6.8%) *	* Calculated in females 2 years post-diagnosis and AMH < 0.5 mg/L, all chemotherapy regimens	Menarchal age median (IQR) 13.0 (11.0; 16.0)

Summary of cohort studies assessing the prevalence of infertility as assumed in female children/adolescents. The studies are sorted by year of publication. Age and duration of follow-up are given as years with mean (SD), or range where such data are available. Abbreviation: Diagnosis: HL Hodgkin lymphoma. Chemotherapy: ABVD Adriamycin (doxorubicin), Bleomycin, Vinblastine, Dacarbazine, COPDAC-28 Cyclophosphamide, Oncovin (vincristine), Prednisone, Dacarbazine; 28-day cycle, DECOPDAC-21 Dacarbazine, Etoposide, Cyclophosphamide, Oncovin (vincristine), Prednisone, Doxorubicin; 21-day cycle, MOPP Mustine (mechlorethamine), Oncovin (vincristine), Procarbazine, Prednisone, OEPA Oncovin (vincristine), Etoposide, Prednisone, Adriamycin (doxorubicin). Parameters: AMH anti-Müllerian hormone. Others: IQR—Interquartile Range. * Presumed infertility parameter calculated after treatment with all chemotherapy regimens.

**Table 5:** Characteristics of the included studies, male children/adolescents.

First Author, Year of Publication	Country	Study Design	Number of Participants of Interest (Males, Adults)	Age of Participants of Interest at Diagnosis/ Therapy (Years, Range)	Age (Years, Mean +/− SD) at Outcome/ Evaluation	Follow-Up After Treatment, Length in Years (Range)	Tumor Type	Chemotherapy, Details	Infertility as Assumed	Infertility Parameter	Comments
Ben Arush et al. 2000 [60]	Israel	Retrospective	8	14.1 (2.1–16.4)	23.3 (14.8–24.3)	7.9 (4.1–17.3)	HL, Stages 1A (12.5%), 2A (37.5%), 2B (25%), 3A (25%)	MOPP, MOPP/ABVD	7/8 (87.5%) *	* Calculated in men with azoospermia, oligozoospermia and/or FSH > 10 U/L, all chemotherapy regimens	Complete cohort (N = 12 HL, N = 8 NHL): N = 9 prepubertal (Tanner I), N = 3 intrapubertal (Tanner II–III), N = 8 postpubertal (Tanner V); Prepubertal status does not protect from treatment-related gonadotoxicity, as severe impairment of spermatogenesis was observed in most patients treated before puberty.
Bordallo et al. 2004 [61]	Brazil	Cross-sectional	18	10 (6–19)	18 (17–23)	3–11	HL	C-MOPP/ABV	15/18 (83.3%) *	* Calculated in men with azoospermia or severe oligozoospermia, all chemotherapy regimens	14 prepubertal, 7 postpubertal out of complete HL cohort
Van den Berg et al. 2004 [57]	The Netherlands	Retrospective	33	Complete cohort of males and females (N = 76): MOPP group: 10.8 (5–14.3); ABVD group: 11.7 (3.8–15.2); ABVD/MOPP group: 13 (5–17.2)	Not specified	Complete cohort of males and females (N = 76): MOPP group: 16.3 (2–24.2); ABVD group: 12.3 (4.9–15.6); ABVD/MOPP group: 5.8 (0.6–11.3)	HL, complete cohort of males and females: Stage I: 39.5%, Stage II: 26.3%, Stage III: 27.6%, Stage IV: 6.6%	MOPP, ABVD, MOPP/ABVD	14/33 (42.4%) *	* Calculated in men with FSH > 10 U/L, all chemotherapy regimens	All prepubertal
Hobbie et al. 2005 [62]	USA	Retrospective	11	13 (6–19)	21 (18–31)	6.5 (1.5–21)	HL	COPP/ABV	9/11 (81.8%) *	* Calculated in men with azoospermia, oligozoospermia, low testosterone < 350 NG/DL and/or FSH > 10 U/L, all chemotherapy regimens	3/11 prepubertal, 8/11 intra- or postpubertal
Van Beek et al. 2007 [11]	The Netherlands	Retrospective	21	11.4 (3.7–15.9)	27 (1.7–42.6)	15.5 (5.6–30.2)	HL, Stage I: 39.3%, II: 30.4%, III: 1.8%, IV: 1.8%, unknown: 1.8%	ABVD or EBVD with or without MOPP	13/21 (61.9%) *	* Calculated in men with azoospermia or oligozoospermia, all chemotherapy regimens	Pubertal status for complete cohort: N = 37 prepubertal, N = 15 pubertal, N = 4 (ages 12.3–13.7 years) no data on pubertal status
Romerius et al. 2010 [63]	Sweden	Retrospective	19	Complete cohort of childhood cancer survivors (N = 129): 10 (0.1–17)	Complete cohort of childhood cancer survivors (N = 129): 29 (20–49)	Complete cohort of childhood cancer survivors (N = 129): 19 (4–36)	HL	Not specified; agents used for complete cohort of childhood cancer survivors: Carmustine, Lomustine, Chlorambucil, Cisplatin, Cyclophosphamide, Melphalan, Procarbazine	10/19 (52.6%) *	* Calculated in men with azoospermia	Other childhood cancers: Leukemias, brain tumors, HL, NHL, testicular cancer, Wilm’s tumor; Pubertal stage: n = 4: age ≤ 10 years (prepubertal, according to the authors); n = 15: age > 10 years (postpubertal)
Rendtorff et al. 2012 [64]	Germany	Retrospective	5	14 (10–17)	27 (24–30)	14 (10–18)	HL	Not specified, only oncological treatment in childhood or adolescence	5/5 (100%) *	* Calculated in men with oligozoospermia or azoospermia	Significantly higher rate of azoospermia/oligozoospermia observed in patients who had been treated during or after puberty
Servitzoglou et al. 2015 [65]	France	Retrospective	45	Complete cohort of HL and NHL (N = 171): 10.8 (2.1–17.3)	Complete cohort of HL and NHL (N = 171): 21.1 (17–30.4)	Complete cohort of HL and NHL (N = 171): 9.3 (2–22.4)	HL	MOPP alone or with ABVD or ABVP; VBVP with OPPA or with COPP	20/45 (44.4%) *	* Calculated in men with FSH level > 10 IU/L, treatment with procarbazine containing chemotherapy	Complete cohort (N = 171): 63.2% prepubertal, 27.5% during puberty, 9.3% postpubertal
Duca et al. 2019 [66]	Italy	Cross-sectional	7	10.3 +/− 4.1	24.1 +/− 5.4	Not specified	HL	Treatment with at least chemotherapy	5/7 (71.4%) *	* Calculated in men with FSH level > 10 IU/L	Subjects aged <10 years probably prepubertal, no further information

Summary of cohort studies assessing the prevalence of infertility as assumed in male children/adolescents. The studies are sorted by year of publication. Age and duration of follow-up are given as years with mean (SD), or range where such data are available. Abbreviation: Diagnosis: HL Hodgkin lymphoma, NHL non-Hodgkin lymphoma. Chemotherapy: ABV Adriamycin (doxorubicin), Bleomycin, Vinblastine, ABVD Adriamycin (doxorubicin), Bleomycin, Vinblastine, Dacarbazine, C-MOPP Cyclophosphamide, Mustine (mechlorethamine), Oncovin (vincristine), Procarbazine, Prednisone, COPP Cyclophosphamide, Oncovin (vincristine), Procarbazine, Prednisone, EBVP Etoposide, Bleomycin, Vinblastine, Prednisone, MOPP Mustine (mechlorethamine), Oncovin (vincristine), Procarbazine, Prednisone, OPPA Oncovin (vincristine), Procarbazine, Prednisone, Adriamycin (doxorubicin), VBVP—Vindesine, Bleomycin, Vinblastine, Prednisone. Parameters: FSH follicle-stimulating hormone. * Presumed infertility parameter calculated after treatment with all chemotherapy regimens.

**Table 6:** Quality assessment using the Joanna Briggs Institute Cohort Study Checklist.

First Author, Year of Publication	(1) Were the Two Groups Similar and Recruited from the Same Population?	(2) Were the Exposures Measured Similarly to Assign People to Both Exposed and Unexposed Groups?	(3) Was the Exposure Measured in a Valid and Reliable Way?	(4) Were Con-Founding Factors Identified?	(5) Were Strategies to Deal with Con-Founding Factors Stated?	(6) Were the Groups/Participants Free of the Outcome at the Start of the Study (or at the Moment of Exposure)?	(7) Were the Outcomes Measured in a Valid and Reliable Way?	(8) Was the Follow-Up Time Reported and Sufficient to Be Long Enough for Outcomes to Occur?	(9) Was Follow-Up Complete, and If Not, Were the Reasons for Loss to Follow-Up Described and Explored?	(10) Were Strategies to Address Incomplete Follow-Up Utilized?	(11) Was Appropriate Statistical Analysis Used?	Total Score	Quality (High: 9–11, Moderate: 6–8; Low: 0–5)
Ben Arush et al. 2000 [60]	0	1	1	1	0	0	1	1	0	0	1	6	moderate
Frias et al. 2003 [49]	1	1	1	1	0	1	1	1	1	0	1	9	high
Van den Berg et al. 2004 [57]	1	1	1	1	0	0	1	1	0	0	1	7	moderate
Bordallo et al. 2004 [61]	0	1	1	0	0	1	1	0	0	0	1	5	low
Behringer et al. 2005 [13]	1	1	1	1	1	1	0	1	0	0	1	8	moderate
Bizet et al. 2012 [50]	0	1	1	0	0	0	1	1	0	0	1	5	low
Hobbie et al. 2005 [62]	0	1	1	1	0	0	1	1	0	0	1	6	moderate
Verschuuren et al. 2006 [28]	1	1	1	0	0	1	1	1	1	0	1	8	moderate
Haukvik et al. 2006 [29]	1	1	1	1	0	1	0	1	1	0	1	8	moderate
Giuseppe et al. 2007 [30]	1	1	1	1	0	1	1	1	1	1	1	10	high
Van der Kaaij et al. 2007 [51]	1	1	1	1	1	1	1	1	0	0	1	9	high
Van Beek et al. 2007 [11]	1	1	1	1	1	1	1	1	0	0	1	9	high
Huser et al. 2008 [31]	1	1	1	1	1	1	1	1	0	0	1	9	high
Blumenfeld et al. 2008 [32]	1	1	1	1	1	1	1	1	1	1	1	11	high
De Bruin et al. 2008 [33]	1	1	1	1	1	1	0	1	1	1	1	10	high
Sieniawski et al. 2008 [52]	1	1	1	1	1	0	1	1	1	0	1	9	high
Nitzschke et al. 2009 [34]	1	1	1	1	0	1	1	1	0	0	1	8	moderate
Kiserud et al. 2009 [9]	1	1	1	1	1	0	1	0	0	1	1	8	moderate
Menon et al. 2009 [53]	0	1	1	0	0	0	1	1	0	0	1	5	low
Behringer et al. 2010 [35]	1	1	1	1	1	1	1	1	1	1	1	11	high
Romerius et al. 2010 [63]	1	1	0	1	1	1	1	1	1	1	1	10	high
Dann et al. 2011 [36]	1	1	1	1	1	1	1	1	1	1	1	11	high
Behringer et al. 2012 [37]	1	1	1	1	1	1	1	1	1	1	1	11	high
Van der Kaaij et al. 2012 [38]	1	1	1	1	1	1	0	1	1	1	1	10	high
Rendtorff et al. 2012 [64]	0	1	1	1	0	1	1	1	0	0	1	7	moderate
Behringer et al. 2013 [14]	1	1	1	1	1	1	1	1	1	1	1	11	high
Swerdlow et al. 2014 [39]	1	1	1	1	1	1	0	1	1	1	1	10	high
Huser et al. 2015 [40]	1	1	1	1	0	1	1	1	1	1	1	10	high
Tomlinson et al. 2015 [54]	0	1	1	1	0	0	1	1	0	0	1	6	moderate
Servitzoglou et al. 2015 [65]	1	1	1	1	1	1	1	1	0	0	1	9	high
Boltezar et al. 2016 [41]	1	1	1	1	0	1	0	1	0	0	1	7	moderate
Paoli et al. 2016 [55]	1	1	1	1	0	0	1	1	0	0	1	7	moderate
Gupta et al. 2016 [58]	0	1	1	0	0	1	1	1	1	1	1	8	moderate
Anderson et al. 2018 [42]	1	1	1	1	1	1	1	1	1	1	1	11	high
Duca et al. 2019 [66]	1	1	0	1	1	1	1	0	0	0	1	7	moderate
Demeestere et al. 2021 [43]	1	1	1	1	1	1	1	1	1	1	1	11	high
Decanter et al. 2021 [44]	1	1	1	1	0	1	1	1	0	0	1	8	moderate
Amzai et al. 2022 [45]	1	1	1	1	0	1	0	1	0	0	1	7	moderate
Laddaga et al. 2022 [56]	1	1	1	1	0	0	1	1	0	0	1	7	moderate
Ciccarone et al. 2023 [46]	1	1	1	1	1	1	1	1	0	0	1	9	high
Flatt et al. 2023 [47]	1	1	1	0	0	1	1	1	1	1	1	9	high
Luong et al. 2023 [48]	1	1	1	0	0	1	0	1	1	1	1	8	moderate
Drechsel et al. 2024 [59]	1	1	1	1	1	1	1	1	0	0	1	9	high

### 3.3. Results of the Meta-Analysis

Overall, 43 studies met the inclusion criteria and were included in the meta-analysis.

### 3.4. Pooled Overall Prevalence of Presumed Infertility After All Chemotherapy Regimens

**Figure 2:**
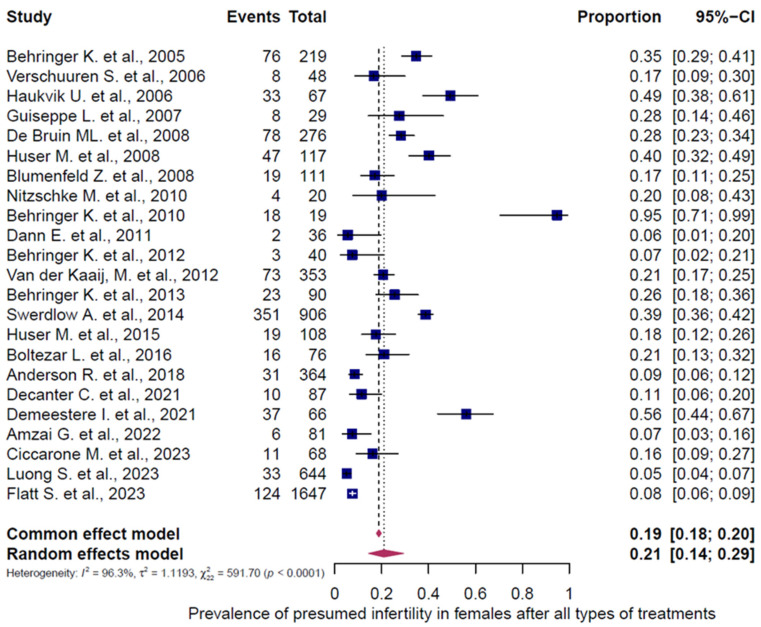
Pooled overall prevalence of presumed infertility in female adults. Forest plot of proportions with 95% confidence intervals (CIs) from studies assessing the prevalence of presumed infertility in adult women after HL chemotherapy. Blue squares represent the proportion in each study, with box size reflecting study weight, and horizontal lines indicating the 95% CI. The pooled prevalence of post-treatment presumed infertility with its 95% CI is highlighted in bold and shown as a pink diamond. Overall estimates are presented for both fixed- and random-effects models. References [13,14,28,29,30,31,32,33,34,35,36,37,38,39,40,41,42,43,44,45,46,47,48].

**Figure 3:**
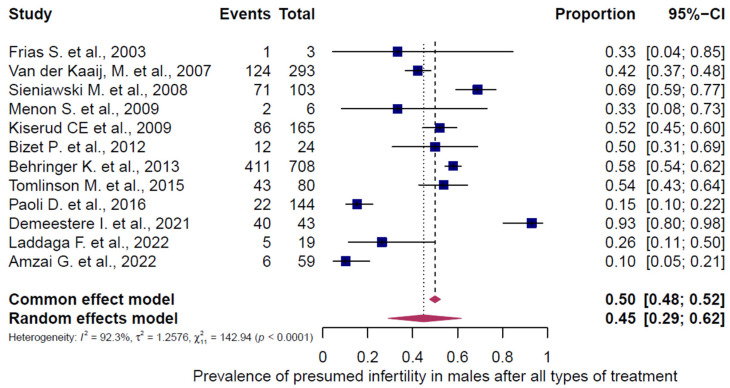
Pooled overall prevalence of presumed infertility in male adults. For details see legend of Figure 2. References [9,14,43,45,49,50,51,52,53,54,55,56].

**Figure 4:**
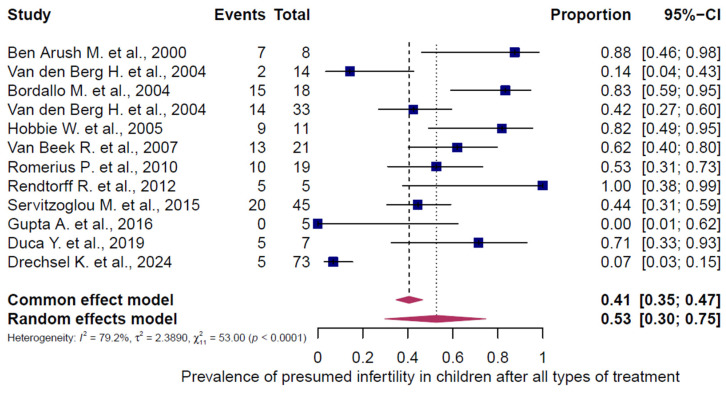
Pooled overall prevalence of presumed infertility in children/adolescents (males and females). For details see legend of Figure 2. References [11,57,58,59,60,61,62,63,64,65,66].

**Figure 5:**
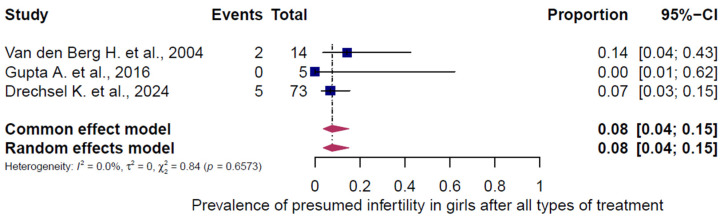
Pooled overall prevalence of presumed infertility in children/adolescents (females). For details see legend of Figure 2. References [57,58,59].

**Figure 6:**
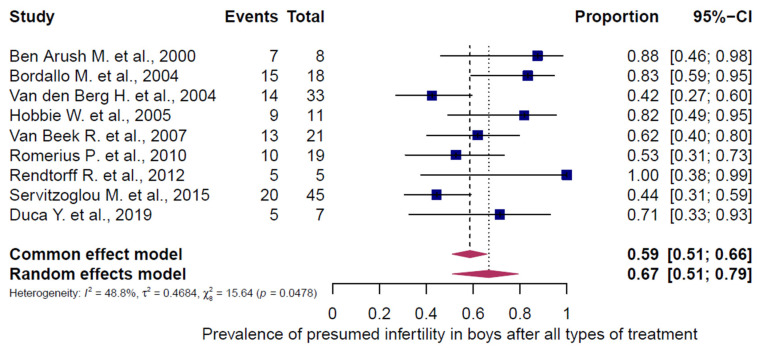
Pooled overall prevalence of presumed infertility in children/adolescents (males). For details see legend of Figure 2. References [11,57,60,61,62,63,64,65,66].

All 43 studies were included in the analysis of presumed infertility prevalence across the reported chemotherapy regimens, comprising 5564 females (5472 adults, 92 children/adolescents) and 1631 males (1464 adults, 167 children/adolescents). The prevalence estimates of the individual studies and the pooled prevalence are presented in Figure 2, Figure 3, Figure 4, Figure 5 and Figure 6. The overall prevalence of presumed infertility was 21% (95% CI: 14–29%) in adult females, 45% (95% CI: 29–62%) in adult males, 53% (95% CI: 30–75%) in children/adolescents overall, 8% (95% CI: 4–15%) in female children/adolescents, and 67% (95% CI: 51–79%) in male children/adolescents. Significant heterogeneity was observed in adult females, adult males, and all children/adolescents combined (I^2^ = 96%, 92%, and 79%; all *p* < 0.0001), whereas no heterogeneity was detected in female children/adolescents (I^2^ = 0%, *p* = 0.6573) and moderate heterogeneity in male children/adolescents (I^2^ = 49%, *p* = 0.0478).

### 3.5. Subgroup Analysis of Presumed Infertility According to Chemotherapy Regimen and Gender

**Figure 7:**
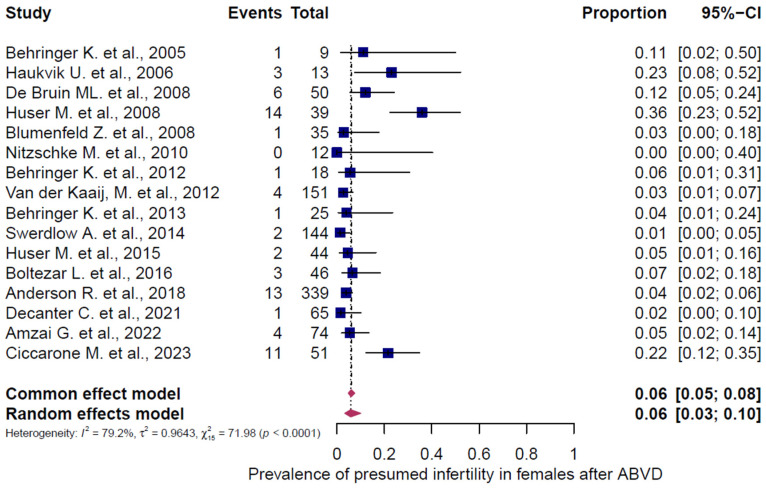
Pooled overall prevalence of presumed infertility in adult females following ABVD chemotherapy. For details see legend of Figure 2. References [13,14,29,31,32,33,34,37,38,39,40,41,42,44,45,46].

**Figure 8:**
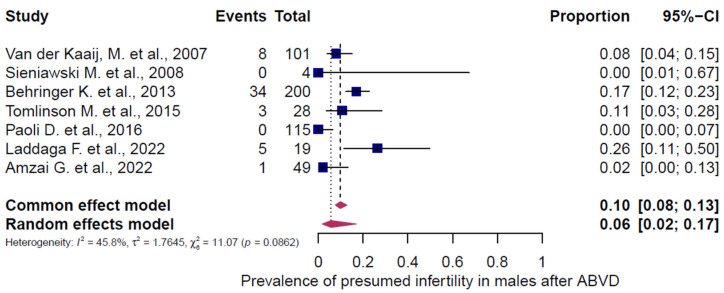
Pooled overall prevalence of presumed infertility in adult males following ABVD chemotherapy. For details see legend of Figure 2. References [14,45,51,52,54,55,56].

**Figure 9:**
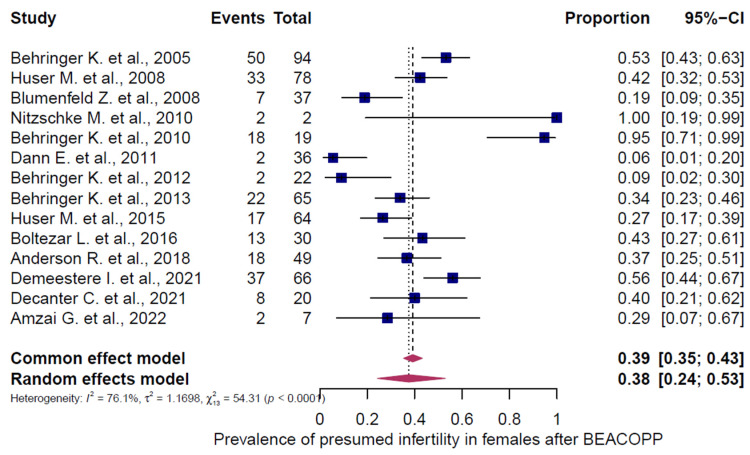
Pooled overall prevalence of presumed infertility in adult females following BEACOPP chemotherapy. For details see legend of Figure 2. References [13,14,31,32,34,35,36,37,40,41,42,43,44,45].

**Figure 10:**
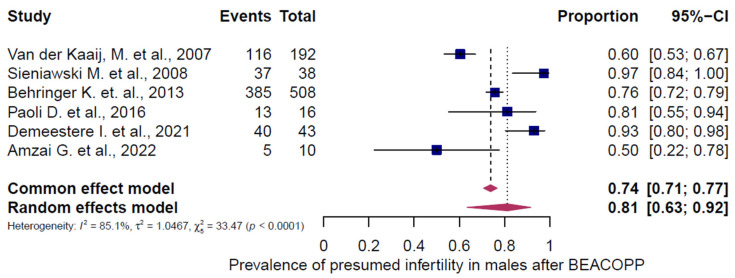
Pooled overall prevalence of presumed infertility in adult males following BEACOPP chemotherapy. For details see legend of Figure 2. References [14,43,45,51,52,55].

**Figure 11:**
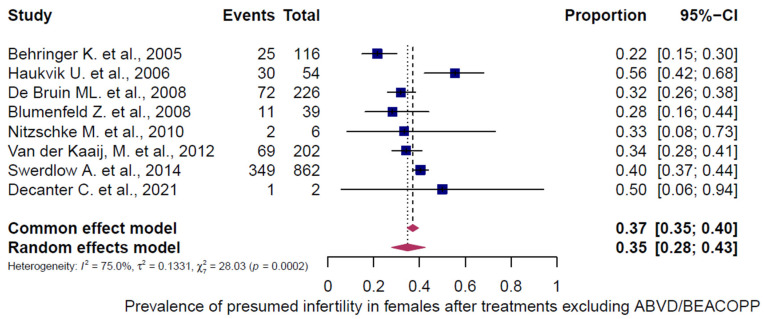
Pooled overall prevalence of presumed infertility in adult females following other chemotherapy regimens. For details see legend of Figure 2, Table 2 and Table 3. References [13,29,32,33,34,38,39,44].

**Figure 12:**
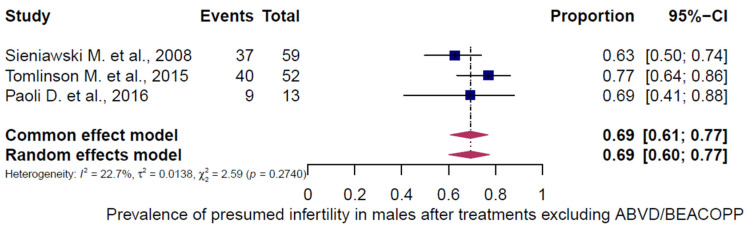
Pooled overall prevalence of presumed infertility in adult males following other chemotherapy regimens. For details see legend of Figure 2, Table 2 and Table 3. References [52,54,55].

Three chemotherapy groups (ABVD, BEACOPP, and others) were evaluated in adult HL patients and stratified by sex (Figure 7, Figure 8, Figure 9, Figure 10, Figure 11 and Figure 12). Presumed infertility prevalence was lowest after ABVD, at 6% in both sexes (95% CI: 3–10% in females [n = 1115], 2–17% in males [n = 516]), with substantial heterogeneity among females (I^2^ = 79%, *p* < 0.0001) and moderate heterogeneity among males (I^2^ = 46%, *p* = 0.0862). Presumed infertility prevalence was highest after BEACOPP, with 38% in females (95% CI: 24–53%, n = 589), for whom the heterogeneity test indicated substantial heterogeneity (I^2^ = 76%, *p* < 0.0001). In males, presumed infertility prevalence reached 81% (95% CI: 63–92%, n = 809), likewise with significant heterogeneity (I^2^ = 85%, *p* < 0.0001). Treatment with other chemotherapy regimens was most frequently based on Meclorethamine, Oncovin (vincristine), Procarbazine, and Prednisone (MOPP); Meclorethamine, Oncovin (vincristine), Procarbazine, Prednisone combined with Adriamycin (doxorubicin), Bleomycin, and Vinblastine (MOPP/ABV); Chlormethine, Lomustine, Vinblastine, Procarbazine, and Prednisone (ChlVPP); or Cyclophosphamide, Oncovin (vincristine), Procarbazine, and Prednisone combined with ABVD (COPP/ABVD).

These regimens were associated with presumed infertility rates of 35% (95% CI: 28–43%) in females (I^2^ = 75%, *p* = 0.0002) and 69% (95% CI: 60–77%) in males (I^2^ = 23%, *p* = 0.2740).

## 4. Discussion

This systematic review and meta-analysis assessed the presumed infertility following chemotherapy for HL, aiming to improve fertility counselling, and is, to the best of our knowledge, the first to stratify results by age, sex, and treatment regimen. Our study revealed an overall pooled prevalence of presumed infertility in HL survivors, with risks in adults for women (21%) and adult men (45%), and in children/adolescents for females (8%) and males (67%). Stratification by chemotherapy regimen shows that the risk of presumed infertility in adults varies by treatment and sex. ABVD is associated with a low risk in both women (6%) and men (6%), BEACOPP confers an intermediate risk in women (38%) and a high risk in men (81%), while other regimens generally carry an intermediate risk in both women (35%) and men (69%).

Numerous studies, including those conducted by the German Hodgkin Study Group (GHSG), have investigated the gonadotoxicity of various therapies in both men and women [8,13,14,35,37] in 562 female HL survivors under 40 years at diagnosis.

Behringer et al. [14] reported that age ≥ 30 years and treatment with BEACOPP were associated with decreased AMH levels, elevated FSH levels, and less regular menstrual cycles, suggesting impaired fertility and highlighting the impact of both age and therapy on post-treatment ovarian function. Similarly, Demesteere et al. [43] found that advanced-stage HL patients (mean age ~27 years) receiving six cycles of BEACOPP had poorer hormone levels and higher rates of premature ovarian insufficiency compared with those treated with a PET-adapted BEACOPP–ABVD regimen, emphasizing the effect of chemotherapy intensity on ovarian function. In contrast secondary amenorrhea occurs infrequently following ABVD therapy without BEACOPP, with a rate of only 3.9% [13].

Our meta-analysis in adult women supports these findings, revealing substantially higher rates of presumed infertility following BEACOPP compared with ABVD treatment.

Other studies in men indicate that chemotherapy primarily affects spermatogenesis, which may already be impaired before treatment, especially in advanced stages, while testosterone production is generally less affected [67].

Behringer et al. [14] found that Inhibin B and FSH levels correlated with chemotherapy intensity, with preserved fertility after early-stage therapy but markedly higher rates of oligozoospermia following six to eight cycles of BEACOPP. Paoli et al. [55] examined sperm parameters and azoospermia rates following different chemotherapy regimens. ABVD caused a temporary reduction in sperm concentration, which normalized within 24 months, suggesting preserved fertility. In contrast, 2–8 cycles of BEACOPP resulted in azoospermia in 50% of cases, and regimens such as COPP, OPP, or MOPP in 85%, consistent with presumed infertility.

Our meta-analysis, incorporating abnormal hormone levels and semen parameters, including azoospermia and oligozoospermia, confirmed higher presumed infertility after BEACOPP, while infertility rates following ABVD aligned with the literature.

The evidence on infertility following chemotherapy for HL in children and adolescents is considerably more limited than in adults, with more data available for male than for female children and adolescents.

Van Beek et al. [11] studied 56 male children/adolescents with HL treated with ABVD/EBVD, with or without alkylating agents (MOPP), evaluating hormone parameters and semen analysis 15.5 years after therapy. Alkylating-agent-containing chemotherapy caused long-term gonadal damage, with 62% of patients showing azoospermia or oligozoospermia, whereas ABVD/EBVD was considerably less gonadotoxic (0% azoospermia/oligozoospermia). Van den Berg et al. [57] studied 17 female children/adolescents with HL. ABVD preserved regular menstrual cycles, whereas alkylating-agent therapy caused irregular cycles in a minority of patients.

These findings largely align with the presumed infertility rates reported in our meta-analyses, indicating a markedly higher risk among male than female survivors of childhood/adolescent HL.

According to current guidelines [4], all patients should be offered fertility preservation prior to potentially gonadotoxic therapy. Based on the results of our study, we recommend an individualized approach tailored to the expected risk of presumed infertility associated with each chemotherapy regimen.

In patients treated with ABVD, where the expected risk of presumed infertility is below 20%, as well as in female children and adolescents, primary (before cancer therapy) fertility preservation is usually not needed. If fertility preservation is not performed, post-treatment follow-up in reproductive medicine should be arranged to assess ovarian reserve and consider secondary (after cancer therapy) preservation options [68].

A presumed infertility risk above 20% is expected with several chemotherapy regimens, including BEACOPP, MOPP, OPPA, COPP, ChlVPP, LOPP, and MVPP. Primary fertility preservation is therefore strongly recommended.

The treatment of newly diagnosed, advanced-stage classical HL is currently undergoing changes based on the results of the HD21 study [7]. BrECADD has been demonstrated to be a more efficacious and better-tolerated alternative to BEACOPP. Across both sexes, FSH recovery four years after therapy was higher following BrECADD than BEACOPP (women: 95% vs. 73%; men: 86% vs. 40%) [8], with a correspondingly higher 5-year incidence of parenthood in BrECADD-treated patients, suggesting substantially lower presumed infertility. Although the BrECADD protocol appears to carry a comparatively lower risk of presumed infertility, long-term data are not yet available and additional studies are needed. Therefore, fertility preservation should be recommended as a precautionary measure for all patients prior to treatment with BrECADD, with counselling provided by specialized reproductive medicine specialists in coordination with the treating oncologists.

Despite rigorous methodology, our study has limitations. Importantly, presumed infertility represents a surrogate-marker-based construct rather than a direct measure of reproductive outcomes such as live birth. Accordingly, many included studies relied on heterogeneous surrogate endpoints, including hormonal parameters, menstrual function, or semen quality, rather than clinically documented infertility. This variability may introduce misclassification and lead to either under- or overestimation of clinically meaningful infertility. Therefore, pooled prevalence estimates should be interpreted as indicators of gonadal dysfunction rather than definitive measures of reproductive incapacity.

A substantial degree of heterogeneity was observed across pooled estimates, particularly in adult and mixed-regimen analyses. This heterogeneity warrants cautious interpretation of the reported prevalence of presumed infertility. Likely contributors include differences in cumulative chemotherapy doses, supportive care measures, duration of follow-up, and variability in outcome definitions across studies. In addition, treatment regimens were not uniformly specified in all cohorts, especially in older studies, further increasing clinical heterogeneity. Nevertheless, patients were treated for Hodgkin lymphoma during eras in which chemotherapy-based regimens constituted standard of care, supporting the assumption of systemic treatment exposure despite incomplete regimen-level detail.

In children and adolescent cohorts, it was often unclear whether treatment occurred pre- or postpubertally, reflecting limited and inconsistent reporting of pubertal status. This limits the assessment of gonadotoxicity, may have influenced infertility risk estimates—particularly among adolescents—and constrains recommendations for fertility preservation.

## 5. Conclusions

This first meta-analysis evaluating the pooled prevalence of presumed infertility after HL chemotherapy, stratified by age, sex, and treatment regimen, provides clinically relevant information to guide fertility counselling. Our results support a differentiated approach: for adult HL patients receiving ABVD and for female children or adolescents, a “wait-and-see” strategy to assess gonadal function post-therapy may be appropriate, with secondary fertility preservation considered if needed. In contrast, primary fertility preservation should be strongly recommended for patients receiving other treatment regimens, particularly those containing alkylating agents such as BEACOPP, and for adolescent males. These findings underscore the importance of personalized counselling and highlight the need for further studies, especially evaluating newer regimens such as BrECADD, to refine risk estimates and optimize fertility preservation strategies.

## Figures and Tables

**Table 1:** Surrogate markers of presumed infertility.

Females	Males
Menstrual cycle disorders Amenorrhea/Oligomenorrhea Hormonal treatment: puberty induction/hormonal replacement therapy	Impairment of sperm quality Azoospermia Oligozoospermia
Hormone levels above the normal range Follicle-stimulating hormone (FSH) Luteinizing hormone (LH)	Hormone levels above the normal range Follicle-stimulating hormone (FSH) Luteinizing hormone (LH)
Premature ovarian insufficiency (POI) Oligo-/amenorrhea for at least 4 months and elevated FSH level > 25 IU/L on two occasions at 4 weeks apart before the age of 40 (ESHRE definition [26]) Or definition depending on the study	Gonadal dysfunction Low testosterone levels Hormonal treatment: testosterone therapy
Low ovarian reserve parameters Anti-Müllerian hormone (AMH) not detectable	Hormone levels below the normal range Inhibin B

## Data Availability

All the data utilized in the study are publicly available and/or contained within the manuscript.

## Supplementary Materials

The following supporting information can be downloaded at: https://www.mdpi.com/article/10.3390/cancers18030425/s1, File S1: Systematic literature search [69].
