# Supplementary material for: Hodgkin Lymphoma—The Effect of Chemotherapy on Gonadal Function and Fertility Is Strongly Related to the Treatment Regimen, Age, and Sex: A Systematic Review and Meta-Analysis
Source: Cancers (Basel). 2026 Jan 28;18(3):425. doi: 10.3390/cancers18030425 (PMC12896784; doi:10.3390/cancers18030425)
Supplement: Supplementary file 1 [file cancers-18-00425-s001.zip › cancers-4078781-supplementary.pdf]

SYSTEMATIC LITERATURE SEARCH

FertiTOX – Effects of Chemotherapy and Radiation on Ovarian Function and Fertility in Patients with Hodgkin Lymphoma

- METHODS -

The literature search for "Effects of Chemotherapy and Radiation on Ovarian Function and Fertility in Patients with Hodgkin Lymphoma" is based on three main concepts to identify potentially relevant publications: 1) Hodgkin lymphoma, 2) chemotherapy and radiotherapy, and 3) fertility.

A search strategy was designed and investigated in MEDLINE, Embase, CENTRAL, and Cochrane Database of Systematic Reviews. A medical information specialist developed an initial search strategy in Embase and tested it against a list of core references to ensure key publications were included. After refinement, the information specialist set up the search strategy for each information source based on database-specific index terms and free text. The free text search included synonyms, acronyms, and similar terms. Using double-negative filters based on MeSH and Emtree terms, animal-only and male-only studies were excluded from the MEDLINE and Embase searches.

The publication years of the results were limited to 2000 to date.

The search was first run on December 8, 2022. A search update was performed on February 5, 2024.

The results were deduplicated using the automated deduplication tool Deduklick[1] [2], the screening tool Covidence detected some few more duplicates. After updating the search, the results from the previous search (2022) that had already been screened were removed from the new results using the "Bramer method"[3].
All references were transferred to the screening tool Covidence[4] for further processing.

---

[1] https://www.risklick.ch/deduklick
[2] Borissov N, et al. Reducing systematic review burden using Deduklick: a novel, automated, reliable, and explainable deduplication algorithm to foster medical research. Syst Rev. 2022 Aug 17;11(1):172. doi: 10.1186/s13643-022-02045-9.
[3] Bramer W, Bain P. Updating search strategies for systematic reviews using EndNote. J Med Libr Assoc. 2017 Jul;105(3):285-289. doi: 10.5195/jmla.2017.183.
[4] https://www.covidence.org/

- OVERVIEW -

**Search date**	**Database searched**	**Platform**	**Dates of database coverage**	**Records**
Feb 5, 2024	MEDLINE ALL	Ovid (Wolters Kluwer)	1946 – Feb 2, 2024	668
Feb 5, 2024	Embase	Ovid (Wolters Kluwer)	1946 – Feb 2, 2024	2309
Feb 5, 2024	Cochrane CENTRAL Database of Clinical Trials	Wiley	1908 – present	114
Feb 5, 2024	Cochrane Database of Systematic Reviews	Wiley	1908 – present	2
**Sum of references**				3093
**Sum of references after deduplication**				**2376**

- DATABASE SEARCH STRATEGIES -

MEDLINE

Ovid MEDLINE(R) ALL <1946 to February 02, 2024>
Search Date: 05/02/2024

1 (Hodgkin* or (malign* adj2 (lymphogranuloma* or granuloma*)) or "Reed-sternberg cell*" or "Sternberg-reed cell*" or "Classic* HD" or "classic* HL" or Reticulolymphosarcom* or germinoblastom*).ti,ab,kf. 75953

2 exp Hodgkin Disease/ or Reed-Sternberg Cells/ 36104

3 or/1-2 85186

4 (chemotherap* or chemo-therap* or chemoradiotherap* or chemo-radiotherap* or adjuvant drug therap* or carcinochemotherap* carcino-chemotherap* or antineoplastic agent* or anti-neoplastic agent* or antineoplastic drug* or anti-neoplastic drug* or antitumo?r agent* or anti-tumo?r agent* or antitumo?r drug* or anti-tumo?r drug* or anticancer* agent* or anti-cancer* agent* or anticancer* drug* or anti-cancer drug* or anticarcinogen* or anti-carcinogen* or anticancerogen* or anti-cancerogen* or ((cancer* or tumo?r* or neoplas*) adj3 treat*)).ti,ab,kf. 894929

5 exp Antineoplastic Agents/ or exp Combined Modality Therapy/ or exp Antineoplastic Combined Chemotherapy Protocols/ or exp chemotherapy, adjuvant/ or

exp Antineoplastic Protocols/ or exp chemoradiotherapy/ or radioimmunotherapy/ 1509804

6 (Radio-therap* or radiotherap* or radiationtherap* or chemoradiotherap* or radiochemotherap* or protontherap* or radiosurg* or radio-surg* or irradiation* or x-ray-therap* or therap* radiolog* or IMRT* or IORT* or radioimmunotherap* or radio-immuno-therap* or ((radiat* or irradiat* or radioisotope* or radio-isotope* or chemoradio or chemo-radio or radiochemo or radio-chemo or proton or x-ray or xray) adj2 (therap* or oncolog* or brachytherap* or brachy-therap*))).ti,ab,kf. 533715

7 exp Radiotherapy/ or exp radiotherapy, adjuvant/ 209919

8 or/4-7 2349586

9 (fertili#ation* or fertility or fertile or fecund* or subfecund* or sub-fecund* or infecund* or infertility or sterility or subfert* or sub-fert* or anovularit* or gonad* or reproductive organ* or reproduction* or gamete-producing gland* or ovarian reserve* or ovary or ovaries or ovarian follicle* or oogenesis or oocyte* or amenorrhea* or premature menopaus* or early menopaus* or climacterium pr?ecox or Gonadotropin* or AMH or Anti-Mu?llerian Hormone* or Antimu?llerian Hormone* or Anti-Mu?llerian Factor* or Mu?llerian Inhibiting Hormone* or mu?llerian inhibitor* or FSH or Follicle Stimulating Hormone* or Folliculostimulating Hormone* or Follitropin or FSH-releasing hormone* or LH-FSH or testis or testes or testicle* or spermatogenes* or sperm* or semen or gametogenes* or hypogonadism* or hypo-gonadism* or "reproductive system*" or azoospermia* or spermatozoon*).ti,ab,kf. 793138

10 exp Fertility/ or exp Infertility/ or exp Gonads/ or Amenorrhea/ or anovulation/ or menopause, premature/ or Reproduction/ or Gametogenesis/ or Spermatozoa/ 381879

11 or/9-10 886085

12 3 and 8 and 11 1176

13 (exp animals/ or exp animal experimentation/ or exp models, animal/ or exp plants/ or exp fungi/) not humans/ 5631279

14 12 not 13 1163

15 limit 14 to yr="2000-current" 668

Link to search:
https://ovidsp.ovid.com/ovidweb.cgi?T=JS&NEWS=N&PAGE=main&SHAREDSEARCHID=6lDt2rEIA6imgiuWYyH0ohQVqI77CzE2uD3lH43sz9Jsb2f2PDu3VmWA0c05ZrWvR

****

EMBASE

Embase <1946 to February 02, 2024>
Search Date: 05/02/2024

1 (Hodgkin* or (malign* adj2 (lymphogranuloma* or granuloma*)) or "Reed-sternberg cell*" or "Sternberg-reed cell*" or "Classic* HD" or "classic* HL" or Reticulolymphosarcom* or germinoblastom*).ti,ab,kf. 109630

2 exp Hodgkin disease/ or Reed Sternberg cell/ 61652

3 or/1-2 128480

4 (chemotherap* or chemo-therap* or chemoradiotherap* or chemo-radiotherap* or adjuvant drug therap* or carcinochemotherap* carcino-chemotherap* or antineoplastic agent* or anti-neoplastic agent* or antineoplastic drug* or anti-neoplastic drug* or antitumo?r agent* or anti-tumo?r agent* or antitumo?r drug* or anti-tumo?r drug* or anticancer* agent* or anti-cancer* agent* or anticancer* drug* or anti-cancer drug* or anticarcinogen* or anti-carcinogen* or anticancerogen* or anti-cancerogen* or ((cancer* or tumo?r* or neoplas*) adj3 treat*)).ti,ab,kf. 1350775

5 exp antineoplastic agent/ or exp multimodality cancer therapy/ or exp cancer chemotherapy/ or exp antineoplastic protocol/ 3198869

6 (Radio-therap* or radiotherap* or radiationtherap* or chemoradiotherap* or radiochemotherap* or protontherap* or radiosurg* or radio-surg* or irradiation* or x-ray-therap* or therap* radiolog* or IMRT* or IORT* or radioimmunotherap* or radio-immuno-therap* or ((radiat* or irradiat* or radioisotope* or radio-isotope* or chemoradio or chemo-radio or radiochemo or radio-chemo or proton or x-ray or xray) adj2 (therap* or oncolog* or brachytherap* or brachy-therap*))).ti,ab,kf. 734725

7 exp cancer radiotherapy/ 341910

8 or/4-7 4107177

9 (fertili#ation* or fertility or fertile or fecund* or subfecund* or sub-fecund* or infecund* or infertility or sterility or subfert* or sub-fert* or anovularit* or gonad* or reproductive organ* or reproduction* or gamete-producing gland* or ovarian reserve* or ovary or ovaries or ovarian follicle* or oogenesis or oocyte* or amenorrhea* or premature menopaus* or early menopaus* or climacterium pr?ecox or Gonadotropin* or AMH or Anti-Mu?llerian Hormone* or Antimu?llerian Hormone* or Anti-Mu?llerian Factor* or Mu?llerian Inhibiting Hormone* or mu?llerian inhibitor* or FSH or Follicle Stimulating Hormone* or Folliculostimulating Hormone* or Follitropin or FSH-releasing hormone* or LH-FSH or testis or testes or testicle* or spermatogenes* or sperm* or semen or gametogenes* or hypogonadism* or hypo-gonadism* or "reproductive system*" or azoospermia* or spermatozoon*).ti,ab,kf. 901576

10 exp fertility/ or exp infertility/ or exp semen analysis/ or exp gonad/ or exp amenorrhea/ or exp early menopause/ or reproduction/ or gametogenesis/ or spermatozoon/ 588544

11 or/9-10 1049853

12 3 and 8 and 11 2952

13 (exp animal/ or exp invertebrate/ or nonhuman/ or animal experiment/ or animal tissue/ or animal model/ or exp plant/ or exp fungus/) not (exp human/ or human tissue/) 7842313

14 12 not 13 2903

15 limit 14 to yr="2000-current" 2309

Link to search:
https://ovidsp.ovid.com/ovidweb.cgi?T=JS&NEWS=N&PAGE=main&SHAREDSEARCHID=456Kk08MgaWu2Zfrt6tCOGvOUWo0TiVt5XAO6Xkanz4oueThFzbZ1Pol5iBrcvZDC

Cochrane Database of Systematic Reviews and Clinical Trials
Search Date: 05/02/2024

#1 (Hodgkin* or (malign* NEAR/2 (lymphogranuloma* or granuloma*)) or (Reed-sternberg NEXT cell*) or (Sternberg-reed NEXT cell*) or (Classic* NEXT hd) or (classic* NEXT hl) or Reticulolymphosarcom* or germinoblastom*):ti,ab,kw 6326

#2 [mh "Hodgkin Disease"] OR [mh ^"Reed-Sternberg Cells"] 1062

#3 #1 or #2 6326

#4 (chemotherap* or chemo-therap* or chemoradiotherap* or chemo-radiotherap* or (adjuvant NEXT drug NEXT therap*) or carcinochemotherap* carcino-chemotherap* or (antineoplastic NEXT agent*) or (anti-neoplastic NEXT agent*) or (antineoplastic NEXT drug*) or (anti-neoplastic NEXT drug*) or (antitumor NEXT agent*) or (antitumour NEXT agent*) or (anti-tumor NEXT agent*) or (anti-tumour NEXT agent*) or (antitumor NEXT drug*) or (antitumour NEXT drug*) or (anti-tumor NEXT drug*) or (anti-tumour NEXT drug*) or (anticancer* NEXT agent*) or (anti-cancer* NEXT agent*) or (anticancer* NEXT drug*) or (anti-cancer NEXT drug*) or anticarcinogen* or anti-carcinogen* or anticancerogen* or anti-cancerogen* or ((cancer* or tumor* or tumour*or neoplas*) NEAR/3 treat*)):ti,ab,kw 124032

#5 [mh "Antineoplastic Agents"] OR [mh "Combined Modality Therapy"] OR [mh "Antineoplastic Combined Chemotherapy Protocols"] OR [mh "chemotherapy,

adjuvant"] OR [mh "Antineoplastic Protocols"] OR [mh "Chemoradiotherapy"] OR [mh ^Radioimmunotherapy] 58244

#6 (Radio-therap* or radiotherap* or radiationtherap* or chemoradiotherap* or radiochemotherap* or protontherap* or radiosurg* or radio-surg* or irradiation* or x-ray-therap* or (therap* NEXT radiolog*) or IMRT? or IORT? or radioimmunotherap* or radio-immuno-therap* or ((radiat* or irradiat* or radioisotope* or radio-isotope* or chemoradio or chemo-radio or radiochemo or radio-chemo or proton or x-ray or xray) NEAR/2 (therap* or oncolog* or brachytherap* or brachy-therap*))):ti,ab,kw 52982

#7 [mh Radiotherapy] OR [mh "Radiotherapy, Adjuvant"] 9961

#8 #4 or #5 or #6 or #7 164493

#9 (fertilization* or fertilisation* or fertility or fertile or fecund* or subfecund* or sub-fecund* or infecund* or infertility or sterility or subfert* or sub-fert* or anovularit* or gonad* or (reproductive NEXT organ*) or reproduction* or (gamete-producing NEXT gland*) or (ovarian NEXT reserve*) or ovary or ovaries or (ovarian NEXT follicle*) or oogenesis or oocyte* or amenorrhea* or (premature NEXT menopaus*) or (early NEXT menopaus*) or (climacterium NEXT praecox) or (climacterium NEXT precox) or Gonadotropin* or AMH or (Anti-Mullerian NEXT Hormone*) or (Anti-Muellerian NEXT Hormone*) or (Antimullerian NEXT Hormone*) or (Antimuellerian NEXT Hormone*) or (Anti-Mullerian NEXT Factor*) oe (Anti-Muellerian NEXT Factor*) or (Mullerian NEXT Inhibiting NEXT Hormone*) or (Muellerian NEXT Inhibiting NEXT Hormone*) or (mullerian NEXT inhibitor*) or (muellerian NEXT inhibitor*) or FSH or (Follicle NEXT Stimulating NEXT Hormone*) or (Folliculostimulating NEXT Hormone*) or Follitropin or (FSH-releasing NEXT hormone*) or LH-FSH or testis or testes or testicle? or spermatogenes* or sperm* or semen or gametogenes* or hypogonadism* or hypo-gonadism* or (reproductive NEXT system*) or azoospermia* or spermatozoon*):ti,ab,kw 43654

#10 [mh Fertility] OR [mh Infertility] OR [mh Gonads] OR [mh ^Amenorrhea] OR [mh ^anovulation] OR [mh ^"menopause, premature"] OR [mh ^Reproduction] OR [mh ^Gametogenesis] OR [mh ^Spermatozoa] 7214

#11 #9 or #10 43726

#12 #3 and #8 and #11 with Cochrane Library publication date Between Jan 2000 and Feb 2024 116

Cochrane Reviews: 2
Trials: 114

Link to search:
https://www.cochranelibrary.com/advanced-search/search-manager?search=7372280
